# Molecular recognition between *Escherichia coli* enolase and ribonuclease E

**DOI:** 10.1107/S0907444910030015

**Published:** 2010-08-13

**Authors:** Salima Nurmohamed, Adam R. McKay, Carol V. Robinson, Ben F. Luisi

**Affiliations:** a Department of Biochemistry, University of Cambridge, 80 Tennis Court Road, Cambridge CB2 1GA, England; b Department of Chemistry, University College London, 20 Gordon Street, London WC1H 0AJ, England; c Department of Chemistry, University of Oxford, South Parks Road, Oxford OX1 3QZ, England

**Keywords:** enolase, RNA degradosome, RNA processing, natively unfolded proteins

## Abstract

In *Escherichia coli* and many other bacterial species, the glycolytic enzyme enolase is a component of the multi-enzyme RNA degradosome, an assembly that is involved in RNA processing and degradation. Enolase is recruited into the degradosome through interactions with a small recognition motif located within the degradosome-scaffolding domain of RNase E. Here, the crystal structure of enolase bound to its cognate site from RNase E (residues 823–850) at 1.9 Å resolution is presented. The structure suggests that enolase may help to organize an adjacent conserved RNA-binding motif in RNase E.

## Abstract 2

The glycolytic enzyme enolase associates with the endoribonuclease RNase E in *Escherichia coli* and many other bacterial species. The crystal structure of the complex reveals the basis for the molecular recognition and provides clues as to the possible function of the interaction.

## 1. Introduction

**Figure 1:**
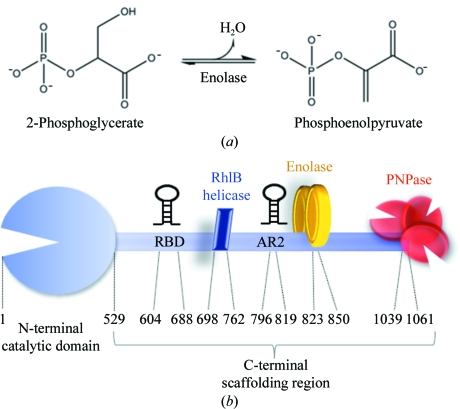
(*a*) Schematic of the reaction catalysed by enolase. The dehydration reaction converts 2-phosphoglycerate into phosphoenolpyruvate and the reverse reaction occurs in gluconeogenesis. (*b*) Schematic cartoon of the *E. coli* RNA degradosome assembly, including the RNA-binding domain (RBD) and arginine-rich domain (AR2).

Enolase (EC 4.2.1.11) is a glycolytic enzyme that is universally conserved in organisms from all domains of life. It catalyses the dehydration of 2-phospho-d-glycerate to form phosphoenolpyruvate and the reverse reaction in gluconeogenesis (Fig. 1 ▶ *a*; Spring & Wold, 1971 ▶). In *Escherichia coli*, approximately one-tenth of the total enolase is associated with the endoribonuclease RNase E in a multi-enzyme complex known as the RNA degradosome (Carpousis, 2007 ▶); the other canonical components of the RNA degradosome are the phosphorolytic exoribonuclease polynucleotide phosphorylase and a DEAD-box RNA helicase, RhlB (Fig. 1 ▶ *b*).

The role of enolase in the degradosome has not been established; however, mutational analyses of RNase E have implicated enolase in the response to phosphosugar stress, which is mediated by the small regulatory RNA SgrS (Morita *et al.*, 2004 ▶). SgrS may be recruited to RNase E through the RNA chaperone Hfq to target the degradation of the transcript encoding the major glucose transporter. DNA microarray analyses suggest that the association of enolase with RNase E in the degradosome affects transcripts that encode enzymes of energy-generating pathways (Bernstein *et al.*, 2004 ▶). It is interesting to note that glycolytic enzymes associate with ribonucleases in *Bacillus subtilus* (Commichau *et al.*, 2009 ▶), which does not have an RNase E homologue. The convergent evolution of complexes composed of ribonucleases and glycolytic enzymes may highlight an important functional role of the interactions (Kang *et al.*, 2010 ▶).

**Figure 2:**
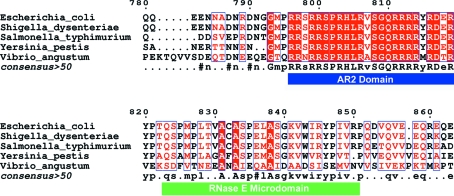
Sequence alignment showing the enolase-recognition site of RNase E. The sequences of enolase-recognition sites from the C-terminal domain of RNase E from representative γ-proteobacterial species (RNase E microdomain, residues 823–850) are indicated by a green bar. The sequence alignment was prepared using *BLAST* and the figure was prepared using *ESPript* (Gouet *et al.*, 1999 ▶). Adjacent to the enolase-binding site is a conserved segment corresponding to residues 798–819 (blue bar) that encompasses the arginine-rich region of RNase E (AR2; see §[Sec sec3]3).

Current evidence indicates that enolase–RNase E recognition is mediated by a small segment of RNase E that is highly conserved amongst γ-proteobacteria (Fig. 2 ▶; Chandran & Luisi, 2006 ▶; Carpousis, 2007 ▶). A recently solved crystal structure of enolase bound to RNase E shows that a minimal binding domain of RNase E (residues 833–850) binds to the inter-protomer groove of an enolase dimer and folds into a compact ‘microdomain’ (Chandran & Luisi, 2006 ▶). The enolase active site is unperturbed in the complex with RNase E, which is consistent with the finding that the interaction does not affect the catalytic activity of enolase (Callaghan *et al.*, 2004 ▶). The residues proceeding 833 in RNase E are also well conserved and it is therefore possible that a longer peptide from the ribonuclease may make additional interactions with enolase (Carpousis, 2007 ▶). Here, we present the crystal structure of enolase bound to its cognate RNase E recognition microdomain, which includes the extended region of conservation from the ribonuclease that was not included in the earlier structural analysis (Chandran & Luisi, 2006 ▶). The new structure is at a resolution of 1.9 Å and reveals that the conserved segment from RNase E does form further interactions with the enolase. The enolase-binding site is physically adjacent to a conserved motif involved in RNA binding and the implications of this proximity for the role of enolase in the degradosome are discussed.

## 2. Materials and methods

### 2.1. Expression and purification of enolase

*E. coli* enolase was overexpressed from a pET11a vector in BL21 (DE3) *E. coli* cells (kindly provided by Dr A. J. Carpousis, CNRS, Toulouse, France) and purified as described previously (Kühnel & Luisi, 2001 ▶). Purified material was stored at 193 K.

### 2.2. Preparation of RNase E recognition microdomain (823–850)

A peptide encompassing the conserved enolase recognition region, corresponding to residues 823–850 of RNase E, was synthesized at the PNAC facility in the Department of Biochemistry, University of Cambridge. This sequence of this microdomain is QSPMPLTVASAAPELASGKVWIRYPIVR. The peptide was reconstituted in 20 m*M* Tris pH 8.0, 20 m*M* MgCl_2_, 150 m*M* NaCl and then desalted using a HiTrap desalting column equilibrated with the same buffer. Peak fractions were stored at 253 K.

### 2.3. Nondissociating mass spectrometry

Purified enolase was mixed with the RNase E microdomain in a 1:1.5 molar ratio and incubated at room temperature for 20 min. The complex was analysed by tandem nondissociating quadrupole–time-of-flight mass spectrometry (QTOF-MS/MS).

### 2.4. Preparation of enolase–RNase E microdomain complex

Complexes of enolase with its RNase E microdomain (residues 823–850) were prepared by mixing the protein and microdomain in a 1:1.5 molar ratio on ice for 20 min. The sample was then used to set up sitting-drop vapour-diffusion crystallization trials using a 1 µl:1 µl volume ratio of complex to reservoir, which was composed of 0.1 *M* HEPES pH 7.0, 1.6 *M* ammonium sulfate. The trays were incubated at 293 K. The crystals obtained were cryoprotected with 1.6 *M* sodium malonate and then flash-frozen in liquid nitrogen.

### 2.5. Data collection, structure determination and refinement

X-ray intensity data were collected on the microfocus beamline ID23-2 at the ESRF in Grenoble, France at 100 K at a wavelength of 0.873 Å. Data were collected from crystals of the enolase–RNase E microdomain complex belonging to the ortho­rhombic space group *P*2_1_2_1_2_1_ at 1.9 Å resolution. The data were processed using the *HKL* package (Otwinowski & Minor, 1997 ▶) and indexed and integrated using *DENZO*. The *CCP*4 suite (Collaborative Computational Project, Number 4, 1994 ▶) was used for further data processing and structure solution. Molecular replacement was performed using *Phaser* (McCoy *et al.*, 2007 ▶) using a dimer from the previously solved *E. coli* enolase structure (Kühnel & Luisi, 2001 ▶) and the RNase E microdomain sequence was built into unbiased density by superimposition with the previously solved enolase–RNase E microdomain structure (Chandran & Luisi, 2006 ▶). The model was built using *Coot* (Emsley & Cowtan, 2004 ▶) and refined using *REFMAC*5 (Murshudov *et al.*, 1997 ▶). Model map inspection was performed using *SFCHECK* (Collaborative Computational Project, Number 4, 1994 ▶) and *RAMPAGE* (Lovell *et al.*, 2003 ▶). Crystallo­graphic and refinement details are given in Table 1 ▶ and the Ramachandran plot analysis is shown in Supplementary Fig. S1^1^. Note that there is one Ramachandran outlier; this is consistent between NCS copies and appears to support an unusual structural element. Structural figures were generated with *PyMOL* (http://www.pymol.org).

## 3. Results

In the present study, enolase was cocrystallized with a longer region of RNase E that contains 28 residues and encompasses the conserved region of RNase E (residues 823–850). The cysteine at position 832 was substituted by alanine to avoid oxidation of the synthetic microdomain. The new crystals were grown under different crystallization conditions from those previously reported and pack in a different lattice (Chandran & Luisi, 2006 ▶). The crystals diffracted to 1.9 Å resolution. The complex with the 28-residue RNase E microdomain belongs to space group *P*2_1_2_1_2_1_, with unit-cell parameters *a* = 103.9, *b* = 110.2, *c* = 160.3 Å, compared with *P*2_1_ for the 15-mer microdomain with pseudo-orthorhombic unit-cell parameters *a* = 77.1, *b* = 124.2, *c* = 96.1 Å, β = 90.6°.

**Figure 3:**
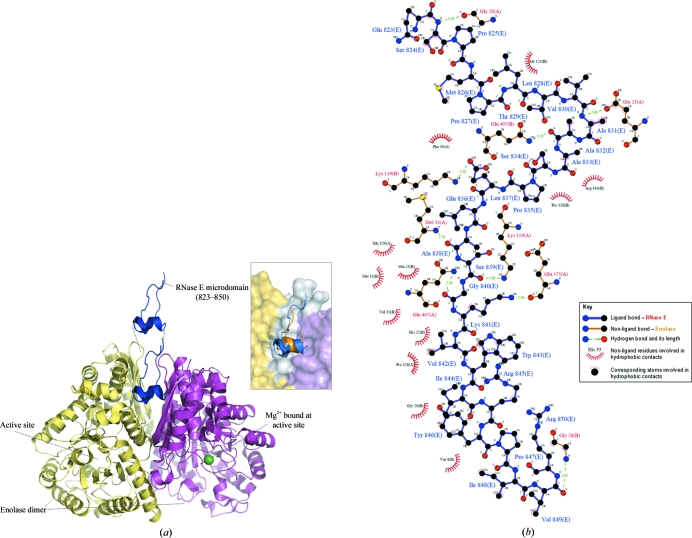
RNase E recognizes the enolase dimer through a microdomain. (*a*) The crystal structure of an *E. coli* enolase dimer (yellow and pink) with RNase E microdomain (blue) corresponding to residues 823–850 (PDB code 3h8a). The inset shows a superimposition of the current and previous *E. coli* enolase structures highlighting the current (blue, PDB code 3h8a) and previous (orange, PDB code 2fym; Chandran & Luisi, 2006 ▶) RNase E-binding sites. (*b*) Schematic summary of the interactions between enolase and its cognate RNase E microdomain (residues 823–850). The figure was prepared using *LIGPLOT* (Wallace *et al.*, 1995 ▶).

**Figure 4:**
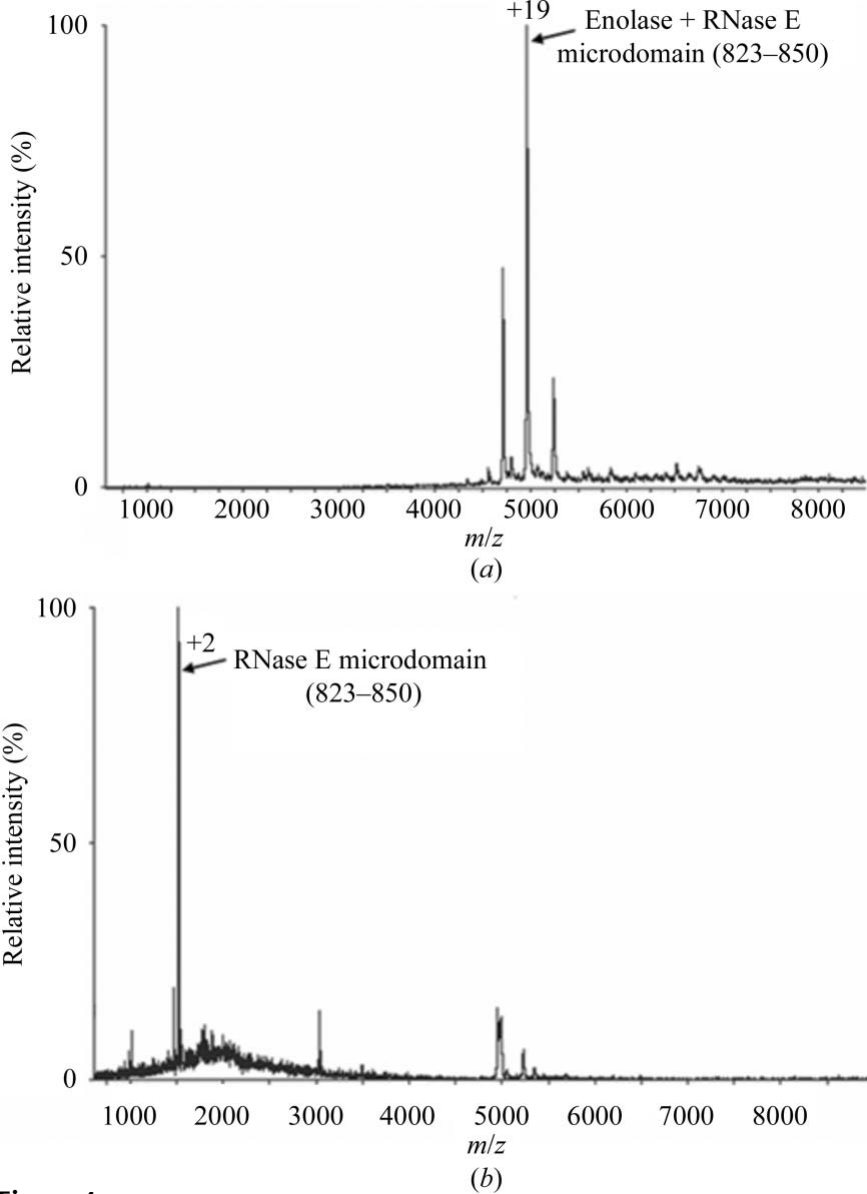
Nanoflow ESI-MS of enolase and RNase E microdomain (823–850) under non­dissociating conditions. (*a*) ESI mass spectrum with a mass corresponding to that of an enolase dimer bound to one molecule of RNase E microdomain (actual mass 94 268 Da, theoretical mass 94 083 Da). (*b*) Tandem mass spectrum of the +19 species in (*a*), showing the presence of the RNase E microdomain (actual mass 3000 Da, theoretical mass 3036 Da).

The crystal structure reveals two independent complexes in the asymmetric unit and the microdomains overlay well for the two complexes. As found in the structure with the shorter version of the recognition site, a single RNase E microdomain is bound in a canyon in the protomer–protomer groove of the dimeric enolase (Fig. 3 ▶). As only a single RNase E microdomain is bound to the dimeric enolase, the interaction is asymmetric. This 1:1 stoichimetry is consistent with the nondissociating mass spectrometry (ESI-MS) data, which confirm that an enolase dimer binds to one RNase E microdomain (residues 823–850; Fig. 4 ▶) and agrees with previous reports using the shorter version of the RNase E microdomain (residues 833–847; Callaghan *et al.*, 2004 ▶; Chandran & Luisi, 2006 ▶). The spectrum of the complex of enolase and the RNase E microdomain (823–850) shows a charge-state series corresponding to a complex of an enolase dimer bound to one RNase E microdomain with a corresponding mass of 94 268.0 (±17.7) Da (Fig. 4 ▶ *b*; theoretical mass of 94 083 Da). The tandem mass spectrum of the +19 charge-state species confirms the presence of the RNase E microdomain (residues 823–850) which appears with a mass smaller than the expected size (Fig. 4 ▶ *b*; theor­etical mass of 3036.66 Da).

The surface area of the peptide buried by the interaction is in the range 330–370 Å^2^, representing weak intermolecular interactions. The binding of the RNase E microdomain has little effect on the structure of the enolase dimer. In comparison to the earlier structure with the shorter RNase E microdomain, the conserved extension shown here continues to span up and out of the inter-protomer groove. The termini of the microdomain are close and may connect without distortion to the remainder of RNase E in the degradosome assembly.

The key interactions between RNase E and enolase are summarized in Fig. 3 ▶(*b*). The RNase E residue Cys832 was substituted by alanine and the C^β^ atom of this amino acid is nestled into a hydrophobic enclosure. It is predicted that cysteine at this position in the native microdomain would be in a favourable location for van der Waals interactions. Oxidation of the cysteine would be likely to disrupt the interaction between RNase E and enolase, but there is no known regulatory mechanism that might involve such a switch.

## 4. Discussion

The enolase-recognizing microdomain is one of the four segments of predicted structural propensity in the C-terminal half of RNase E (Callaghan *et al.*, 2004 ▶). Other microdomains in RNase E mediate interactions with the cytoplasmic membrane (Khemici *et al.*, 2008 ▶), with the RhlB helicase and RNA (Chandran *et al.*, 2007 ▶) and with polynucleotide phosphorylase (Nurmohamed *et al.*, 2009 ▶). The remaining portions of this domain are predicted to be natively un­structured. Here, we have described high-resolution crystallo­graphic studies of *E. coli* enolase in complex with its cognate recognition ‘microdomain’ of RNase E. Our crystal structure of the complex confirms that one *E. coli* RNase E microdomain binds asymmetrically to the inter-protomer groove of enolase that is formed by the surface of β-sheets on the periphery of the triose isomerase-like (TIM) barrel core of the protomer. The 27-residue enolase-recognition segment is conserved in RNases E from many other bacteria. An identical sequence is found in RNase E from *Shigella* sp. and there is a single Leu-to-Met substitution in *Salmonella* sp. (Fig. 2 ▶). A recent study of RNase E from *Vibrio angustum* confirms that it interacts directly with enolase through a segment that is similar in sequence to the microdomain studied here (Fig. 2 ▶; Erce *et al.*, 2009 ▶).

The RNase E microdomain is adjacent to a highly conserved segment corresponding to residues 798–819 (Fig. 2 ▶). This conserved portion of RNase E fully encompasses the arginine-rich segment (AR2) that has been implicated in RNA binding (Carpousis, 2007 ▶). The proximity of the AR2 motif to the free N-terminal end of the enolase-binding microdomain suggests that the AR2 segment could be in a position to make additional contacts with the surface of enolase. Using the *FUGUE* server, which identifies structural homologues based on patterns of environment-dependent substitution propensities (http://tardis.nibio.go.jp/fugue), the RNase E AR2 region is predicted to have a very weak match (*Z* score 4.21) to the Pcf11 protein in the Pcf11–Clp1 polyadenylation factor complex (PDB code 2npi; Noble *et al.*, 2007 ▶). Pcf11 is a short peptide ‘microdomain’ with little globular character and it snakes over the surface of the Clp1 in the cognate complex. We suggest that there may be an analogous interaction between the AR2 microdomain and the surface of enolase. Such an interaction could help to present the AR2 peptide for RNA binding. A structural role of enolase in indirectly facilitating RNA binding would account for its function in the RNA degradosome. This hypothesis awaits experimental verification.

The association of enolase with the degradosome has been shown to affect transcripts of energy-generating metabolism and response to phosphosugar stress (Bernstein *et al.*, 2004 ▶; Morita *et al.*, 2004 ▶; Carpousis, 2007 ▶). It is well established that the degradative machineries in *E. coli* are required for normal mRNA turnover and that they play roles in the decay of transcripts encoding enzymes of energy-generating pathways (Bernstein *et al.*, 2004 ▶). These and other findings suggest that RNA degradation and central metabolism are somehow linked, but the nature of the connection is not presently clear. Further studies may clarify whether degradosome-bound enolase contributes to potential communication between cellular metabolic status and post-transcriptional gene regulation.

## Supplementary Material

PDB reference: enolase–RNase E recognition domain complex, 3h8a

Supplementary material file. DOI: 10.1107/S0907444910030015/hv5161sup1.pdf

## Figures and Tables

**Table 1:** Crystallographic data and refinement summary for the enolase crystal structure (PDB code 3h8a) Values in parentheses are for the last shell.

Space group	*P*2_1_2_1_2_1_
Unit-cell parameters (Å)	*a* = 103.9, *b* = 110.2, *c* = 160.3
Crystallization conditions	0.1 *M* HEPES pH 7.0, 1.6 *M* (NH_4_)_2_SO_4_
Resolution (Å)	24.7–1.90 (1.97–1.90)
Light source	ESRF ID23-2
Wavelength (Å)	0.873
No. of unique reflections	144255
Multiplicity	3.8 (3.1)
Completeness (%)	99.3 (95.1)
〈*I*/σ(*I*)〉	24.4 (2.4)
*R*_merge_ (%)	8.1 (51.2)
Wilson *B* factor (Å^2^)	20.3
Refinement	
Resolution (Å)	24.7–1.9
*R* factor	0.180
*R*_free_	0.226
No. of reflections used	97645
Total No. of atoms	14359
Total No. of amino-acid residues	1774

## Footnotes

1 Supplementary material has been deposited in the IUCr electronic archive (Reference: HV5161). Services for accessing this material are described at the back of the journal.
