# Supplementary figures and images for: Molecular recognition between Escherichia coli enolase and ribonuclease E
Source: Acta Crystallogr D Biol Crystallogr. 2010 Aug 13;66(Pt 9):1036–40. doi: 10.1107/S0907444910030015 (PMC2935283; doi:10.1107/S0907444910030015)

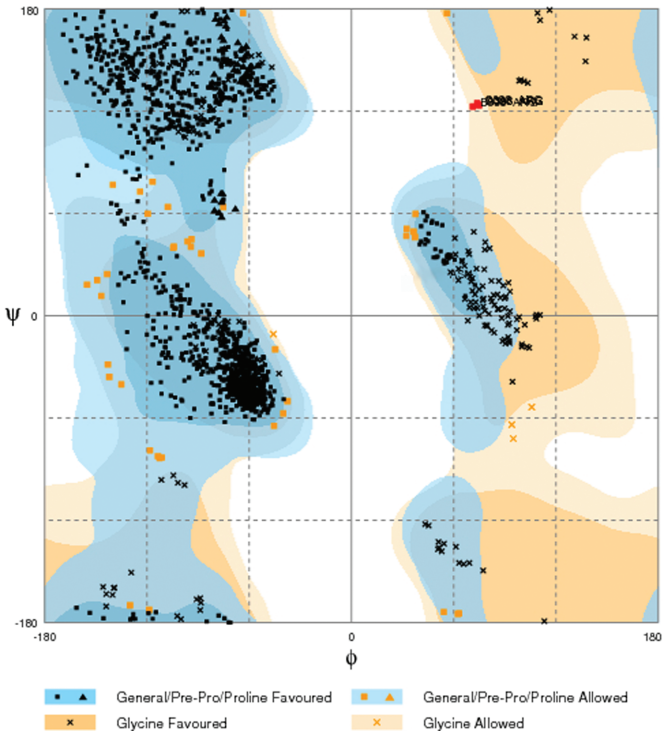

Supplement: Supplementary file 1 [file d-66-01036-sup1.pdf]
